# Loss-of-function genomic variants highlight potential therapeutic targets for cardiovascular disease

**DOI:** 10.1038/s41467-020-20086-3

**Published:** 2020-12-18

**Authors:** Jonas B. Nielsen, Oren Rom, Ida Surakka, Sarah E. Graham, Wei Zhou, Tanmoy Roychowdhury, Lars G. Fritsche, Sarah A. Gagliano Taliun, Carlo Sidore, Yuhao Liu, Maiken E. Gabrielsen, Anne Heidi Skogholt, Brooke Wolford, William Overton, Ying Zhao, Jin Chen, He Zhang, Whitney E. Hornsby, Akua Acheampong, Austen Grooms, Amanda Schaefer, Gregory J. M. Zajac, Luis Villacorta, Jifeng Zhang, Ben Brumpton, Mari Løset, Vivek Rai, Pia R. Lundegaard, Morten S. Olesen, Kent D. Taylor, Nicholette D. Palmer, Yii-Der Chen, Seung H. Choi, Steven A. Lubitz, Patrick T. Ellinor, Kathleen C. Barnes, Michelle Daya, Nicholas Rafaels, Scott T. Weiss, Jessica Lasky-Su, Russell P. Tracy, Ramachandran S. Vasan, L. Adrienne Cupples, Rasika A. Mathias, Lisa R. Yanek, Lewis C. Becker, Patricia A. Peyser, Lawrence F. Bielak, Jennifer A. Smith, Stella Aslibekyan, Bertha A. Hidalgo, Donna K. Arnett, Marguerite R. Irvin, James G. Wilson, Solomon K. Musani, Adolfo Correa, Stephen S. Rich, Xiuqing Guo, Jerome I. Rotter, Barbara A. Konkle, Jill M. Johnsen, Allison E. Ashley-Koch, Marilyn J. Telen, Vivien A. Sheehan, John Blangero, Joanne E. Curran, Juan M. Peralta, Courtney Montgomery, Wayne H-H Sheu, Ren-Hua Chung, Karen Schwander, Seyed M. Nouraie, Victor R. Gordeuk, Yingze Zhang, Charles Kooperberg, Alexander P. Reiner, Rebecca D. Jackson, Eugene R. Bleecker, Deborah A. Meyers, Xingnan Li, Sayantan Das, Ketian Yu, Jonathon LeFaive, Albert Smith, Tom Blackwell, Daniel Taliun, Sebastian Zollner, Lukas Forer, Sebastian Schoenherr, Christian Fuchsberger, Anita Pandit, Matthew Zawistowski, Sachin Kheterpal, Chad M. Brummett, Pradeep Natarajan, David Schlessinger, Seunggeun Lee, Hyun Min Kang, Francesco Cucca, Oddgeir L. Holmen, Bjørn O. Åsvold, Michael Boehnke, Sekar Kathiresan, Goncalo R. Abecasis, Y. Eugene Chen, Cristen J. Willer, Kristian Hveem

**Affiliations:** 1 Department of Internal Medicine: Cardiology, University of Michigan, Ann Arbor, MI USA; 2 K.G. Jebsen Center for Genetic Epidemiology, Department of Public Health and Nursing, Faculty of Medicine and Health Sciences, Norwegian University of Science and Technology, NTNU, Trondheim, Norway; 3 Program in Medical and Population Genetics, Broad Institute of Harvard and MIT, Cambridge, MA USA; 4 Analytic and Translational Genetics Unit, Massachusetts General Hospital, Boston, MA USA; 5 Stanley Center for Psychiatric Research, Broad Institute of Harvard and MIT, Cambridge, MA USA; 6 Department of Computational Medicine and Bioinformatics, University of Michigan, Ann Arbor, MI USA; 7 Department of Biostatistics, University of Michigan School of Public Health, Ann Arbor, MI USA; 8 Center for Statistical Genetics, University of Michigan School of Public Health, Ann Arbor, MI USA; 9 Istituto di Ricerca Genetica e Biomedica, Consiglio Nazionale delle Ricerche (CNR), Monserrato, Cagliari, Italy; 10 Department of Human Genetics, University of Michigan, Ann Arbor, MI USA; 11 Department of Dermatology, St. Olav’s Hospital, Trondheim University Hospital, Trondheim, Norway; 12 Laboratory for Molecular Cardiology, Department of Cardiology, Centre for Cardiac, Vascular, Pulmonary and Infectious Diseases, Copenhagen University Hospital Rigshospitalet, Copenhagen, Denmark; 13 Department of Biomedical Sciences, Faculty of Medicine and Health Sciences, University of Copenhagen, Copenhagen, Denmark; 14 The Institute for Translational Genomics and Population Sciences, Department of Pediatrics and Los Angeles Biomedical Research Institute, Harbor-UCLA, Torrance, CA USA; 15 Department of Biochemistry, Wake Forest School of Medicine, Winston-Salem, NC USA; 16 Cardiovascular Research Center, Massachusetts General Hospital, Boston, MA USA; 17 Colorado Center for Personalized Medicine, School of Medicine, University of Colorado, Aurora, CO USA; 18 Channing Division of Network Medicine, Department of Medicine Brigham and Women’s Hospital, Boston, MA USA; 19 Harvard Medical School, Boston, MA USA; 20 Department of Pathology and Laboratory Medicine, Larner College of Medicine, University of Vermont, Burlington, VT USA; 21 Department of Biochemistry, Larner College of Medicine, University of Vermont, Burlington, VT USA; 22 Department of Medicine, Boston University School of Medicine, Boston, MA 02118 USA; 23 Framingham Heart Study, Framingham, MA USA; 24 Department of Biostatistics, Boston University School of Public Health, Boston, MA USA; 25 GeneSTAR Research Program, Department of Medicine, Johns Hopkins School of Medicine, Baltimore, MD USA; 26 Department of Epidemiology, School of Public Health, University of Michigan, Ann Arbor, MI USA; 27 Survey Research Center, Institute for Social Research, University of Michigan, Ann Arbor, MI USA; 28 The University of Alabama at Birmingham, Birmingham, AL USA; 29 23andMe, Inc., Sunnyvale, CA USA; 30 Deans Office, College of Public Health, University of Kentucky, Lexington, KY USA; 31 Department of Physiology and Biophysics, University of Mississippi Medical Center, Jackson, MS USA; 32 Jackson Heart Study, Jackson, MS USA; 33 Department of Medicine, University of Mississippi Medical Center, Jackson, MS USA; 34 Center for Public Health Genomics, University of Virginia, Charlottesville, VA USA; 35 BloodWorks Northwest, University of Washington, Seattle, WA USA; 36 Duke Molecular Physiology Institute, Duke University Medical Center, Durham, NC USA; 37 Department of Medicine, Duke University Medical Center, Durham, NC USA; 38 Department of Pediatrics, Division of Hematology/Oncology, Baylor College of Medicine, Houston, TX USA; 39 Department of Human Genetics, University of Texas Rio Grande Valley School of Medicine, Brownsville, TX USA; 40 South Texas Diabetes and Obesity Institute, University of Texas Rio Grande Valley School of Medicine, Brownsville, TX USA; 41 Department of Genes and Human Disease, Oklahoma Medical Research Foundation, Oklahoma, OK USA; 42 Division of Endocrinology and Metabolism, Department of Internal Medicine, Taichung Veterans General Hospital, Taichung, Taiwan; 43 Institute of Population Health Sciences, National Health Research Institutes, Miaoli, Taiwan; 44 Division of Biostatistics, Washington University School of Medicine, St. Louis, MO USA; 45 University of Pittsburgh School of Medicine, Pittsburgh, PA USA; 46 University of Illinois at Chicago, Chicago, IL USA; 47 Division of Public Health Sciences, Fred Hutchinson Cancer Research Center, Seattle, WA USA; 48 Department of Epidemiology, University of Washington, Seattle, WA USA; 49 Division of Endocrinology, Diabetes and Metabolism, Ohio State University, Columbus, OH USA; 50 Division of Pharmacogenomics University of Arizona, Tucson, AR USA; 51 Division of Genetics, Genomics and Precision Medicine, Department of Medicine, University of Arizona, Tucson, AR USA; 52 Institute of Genetic Epidemiology, Department of Genetics and Pharmacology, Medical University of Innsbruck, Innsbruck, Austria; 53 Institute for Biomedicine, Eurac Research, Bolzano, Italy; 54 Department of Anesthesiology, University of Michigan, Ann Arbor, MI USA; 55 Center for Genomic Medicine, Massachusetts General Hospital, Harvard Medical School, Boston, MA USA; 56 Laboratory of Genetics, National Institute on Aging, US National Institutes of Health, Baltimore, MD USA; 57 Dipartimento di Scienze Biomediche, Università degli Studi di Sassari, Sassari, Italy; 58 HUNT Research Centre, Department of Public Health and Nursing, Norwegian University of Science and Technology, Levanger, Norway; 59 Department of Endocrinology, St. Olavs Hospital, Trondheim University Hospital, Trondheim, Norway; 60 Broad Institute, Cambridge, MD USA; 61 Regeneron Pharmaceuticals, Tarrytown, NY USA; 62 Levanger Hospital, Nord-Trøndelag Hospital Trust, Levanger, Norway

**Keywords:** Computational biology and bioinformatics, Genome-wide association studies, Cardiovascular diseases

## Abstract

Pharmaceutical drugs targeting dyslipidemia and cardiovascular disease (CVD) may increase the risk of fatty liver disease and other metabolic disorders. To identify potential novel CVD drug targets without these adverse effects, we perform genome-wide analyses of participants in the HUNT Study in Norway (n = 69,479) to search for protein-altering variants with beneficial impact on quantitative blood traits related to cardiovascular disease, but without detrimental impact on liver function. We identify 76 (11 previously unreported) presumed causal protein-altering variants associated with one or more CVD- or liver-related blood traits. Nine of the variants are predicted to result in loss-of-function of the protein. This includes *ZNF529*:p.K405X, which is associated with decreased low-density-lipoprotein (LDL) cholesterol (P = 1.3 × 10^−8^) without being associated with liver enzymes or non-fasting blood glucose. Silencing of *ZNF529* in human hepatoma cells results in upregulation of LDL receptor and increased LDL uptake in the cells. This suggests that inhibition of *ZNF529* or its gene product should be prioritized as a novel candidate drug target for treating dyslipidemia and associated CVD.

## Abstract (web summary)

Drugs targeting cardiovascular disease (CVD) can have negative consequences for liver function. Here, the authors combine genome wide analyses on 69,479 individuals to identify loss-of-function variants with beneficial effects on CVD-related traits without negative impacts on liver function.

## Introduction

Cardiovascular disease (CVD) – in particular cerebrovascular and ischemic heart diseases – is the leading cause of death globally^1^. Lowering circulating lipids is an important treatment strategy to reduce risk^2^. However, some pharmaceutical mechanisms of lipid lowering and CVD risk reduction may unfortunately increase risk of fatty liver disease or other metabolic disorders^3–6^.

The vast majority of novel candidate drugs that enter clinical testing fail to demonstrate sufficient safety and efficacy to gain regulatory approval. This is largely due to poor predictive value of preclinical models of disease along with a lack of knowledge about the long-term consequences of targeting specific biological processes in humans^7^. It has been estimated, however, that drugs with genetic support of efficacy are twice as likely to have success in clinical testing^8^.

We aim to identify novel candidate pharmaceutical strategies for CVD risk reduction that, importantly, are unlikely to increase the risk of liver disease, diabetes, or other metabolic disorders. To attain this, we conduct a large data-driven genomic discovery effort to identify presumed causal protein-altering variants with impact on lipids and other liver-related blood traits. In particular, we are interested in identifying presumed causal protein-altering variants associated with a more favorable lipid profile without being associated with elevated liver enzymes or vice versa.

We analyze 9 liver-related blood traits in close to 70,000 participants in the Trøndelag Health (HUNT) Study. The HUNT Study is a large population-based health survey conducted in a geographically confined region in Norway^9^. The examined traits are related to: (i) blood lipid levels which impact cardiovascular, neurological and eye-related diseases: total cholesterol (TC), low-density lipoprotein cholesterol (LDL-C), high-density lipoprotein cholesterol (HDL-C) and triglyceride (TG) levels; (ii) C-reactive protein (CRP; only values <15 mg/L were included) which is predictive of cardiovascular disease^10^; and (iii) enzymes which primarily reflect liver function: alanine aminotransferase (ALT), aspartate aminotransferase (AST), alkaline phosphatase (ALP) and gamma-glutamyltransferase (GGT).

To maximize chances of discovery of presumed causal protein-altering variants associated with the 9 blood traits, we combine a number of genomic approaches, including (i) low-coverage (5x) whole-genome sequencing (WGS) of a subsample of HUNT Study participants (*N* = 2202) to identify region-specific rare variants, (ii) targeted genotyping, also including rare region-specific variants identified by WGS, (iii) deep genotype imputation based on the TOPMed multi-ethnic reference panel consisting of 60,039 deeply sequenced genomes^11^, (iv) genome-wide association analyses (GWAS) in up to 69,479 HUNT Study participants (ranging from 21,528 for AST to 69,479 for TG), followed by (v) stepwise conditional analyses and, for the purpose of further fine-mapping loci identified in HUNT, we perform (vi) trans-ancestry meta-analyses in up to 203,476 people of Norwegian, Japanese, and Sardinian ancestry (ranging from 128,794 for CRP to 203,476 for TC) (see Supplementary Fig. 1 for a study design overview).

We assume any protein-altering variant to be likely causally related to the trait of interest if the variant (i) was the most statistically significant variant (lowest P) in a genomic region (i.e., the locus index variant) in any of the GWAS or (ii) if the variant was independently associated with the trait of interest in GWAS stepwise conditional analyses.

## Results

### Genomic discovery of presumed causal protein-altering variants

We imputed 26 million genomic variants with sufficient quality and at least 10 minor allele copies into 69,479 participants in the HUNT Study (Supplementary Data 1). Using a linear mixed model^12^ to account for relatedness among study participants, we tested for genome-wide association (*P* < 5 × 10^−8^) with 9 liver-related blood traits. We identified 201 genomic regions (i.e., loci) associated with one or more of the traits. At 24 of the 201 loci, the locus index variant alters (*n* = 22) or results in loss-of-function (LoF) (*n* = 2) of the protein. We consider these 24 variants as presumed causally related to the trait of interest (Supplementary Fig. 2 and Supplementary Data 2). Stepwise conditional analyses resulted in identification of an additional 150 independently associated variants within the 201 loci. These include 28 additional protein-altering variants, hereof 2 LoF variants, which are significantly and independently associated with one or more of the liver-related blood traits (Supplementary Data 3).

For the purpose of further fine-mapping of loci and identification of additional presumed causal protein-altering variants, we performed trans-ancestry meta-analyses by combining summary statistics based on the primary discovery effort in HUNT with additional GWAS statistics from Sardinia (SardiNIA cohort)^13^ and Japan (Biobank Japan)^14^. The combined meta-analyses comprised up to 203,476 participants (*N* range 128,794 for CRP to 203,476 for TC) and 31.5 million unique variants (*n* range 24.7–31.5 million) (Supplementary Fig. 3). The analyses resulted in identification of an additional 86 loci and 351 independent variants, including 13 presumed causal protein-altering variants. One of the protein-altering variants comprise a previously reported LoF variant in *HBB* (p.Q40X)^15^ associated with decreased TC (Table 1 and Supplementary Data 4).

**Table 1:** Loss-of-function variants associated with liver-related blood traits.

Gene variant	Chr:pos	Minor/major allele	rs ID	MAF % (MAC)	Rsq	Trait	*N*	Beta SD	SE	P	Discovery method	Novelty of variant-trait association
*APOB* p.K1813X	2:21011431	A/T	–	0.009 (10)	–	LDL-C	56815	−2.63	0.35	1.1 × 10^−13^	Custom array – predicted nonsense	Novel (in known LDL-C locus; known gene^17,18^)
*APOB* p.R1333X	2:21013379	A/G	rs121918383	0.009 (10)	–	LDL-C	55383	−2.80	0.35	2.5 × 10^−15^	Custom array – predicted nonsense	Novel (in known LDL-C locus; known gene^17,18^, variant previously linked to familial hypobetalipoproteinemia^53^)
*APOB* p.W3087X	2:21007608	T/C	rs745457003	0.011 (12)	–	LDL-C	55383	−2.79	0.32	5.1 × 10^−18^	Custom array – predicted nonsense	Novel (in known LDL-C locus; known gene^17,18^)
*GPLD1* p.V815Sfs*46	6:24429112	C/CT	rs573778305	0.85	0.97	ALP	48578	−0.87	0.039	2.2 × 10^−107^	HUNT locus index variant (imputed from TOPMed)	Novel (in known ALP locus^14^; gene experimentally linked to liver disease^54^)
*HBB* p.Q40X	11:5226774	A/G	rs11549407	4.81	–	TC	5937	−0.48	0.048	5.4 × 10^−23^	Trans-ancestry meta-analysis index variant	Known (previously associated with beta thalassemia^15^, which again is known to be associated with decreased TC^55^)
*LIPC* p.G247Afs*11	15:58545904	ACG/A	rs749932377	0.16 (221)	0.88	HDL-C	69214	0.58	0.081	1.1 × 10^−9^	HUNT conditional analysis (imputed from TOPMed)	Novel (in known HDL-C locus;^17,18^ variant report in carrier with mixed hyperlipidemia but who also carried APOB variants^56^)
*LPL* p.S474X	8:19962213	G/C	rs328	11.9	1.00	TG	180981	−0.17	0.0057	1.3 × 10^−196^	Trans-ancestry meta-analysis index variant	Known variant at known locus^17,18^, experimentally shown to result in gain-of-function of LPL^19^.
*SLC22A1* p.D426Pfs*28	6:160139865	C/CTGGTAAGT	rs113569197	39.3	0.99	LDL-C	67429	−0.05	0.0060	3.3 × 10^−9^	HUNT conditional analysis (imputed from TOPMed)	Novel (in known LDL-C locus^17,18^)
*ZNF529* p.K405X	19:36547291	A/T	rs1376217616	0.099 (110)	–	LDL-C	55383	−0.60	0.11	1.3 × 10^−8^	Custom array – observed in low-pass genomes	Novel

Reported allele frequencies are based on the HUNT population except for HBB p.Q40X which was based on the SardiNIA dataset since it was only present there.

*Chr* Chromosome, *pos* position human genome build hg38, *MAF* Minor allele frequency, *MAC* minor allele count, *Rsq* imputation r2 in HUNT, *SD* standard deviation, *SE* standard error of the beta, *P* *P* value, *LDL-C* Low-density lipoprotein cholesterol, *ALP* Alkaline phosphatase, *TC* Total cholesterol, *HDL-C* High-density lipoprotein cholesterol, *TG* Triglyceride.

To identify rare HUNT-specific presumed causal protein-altering variants, we performed genome-wide association testing for the 9 liver-related blood traits in up to 57,060 HUNT Study participants (*N* range 15,520 for AST to 57,060 for TG) based on directly genotyped variants that were included as custom content on the array and not part of the primary GWAS (see Materials and Methods for details). In brief, variants were selected for custom content genotyping if they were (i) identified by HUNT-specific low-coverage (5x) WGS (*N* = 2202 HUNT Study participants), (ii) observed in Norwegian clinics for familial hypercholesterolemia, or (iii) not-previously-observed variants predicted to introduce a premature stop codon in any of 56 genes in which protein-altering variants are deemed clinically actionable by The American College of Medical Genetics and Genomics (ACMG56)^16^. This resulted in identification of an additional 11 protein-altering variants, including 4 LoF variants, which are associated with one or more of the 9 traits. Five of the 11 variants originated from region-specific WGS and all variants are rare (ranging from 1 in 178 to 1 in 6313 individuals) (Table 1 and Supplementary Data 5).

**Fig. 1:**
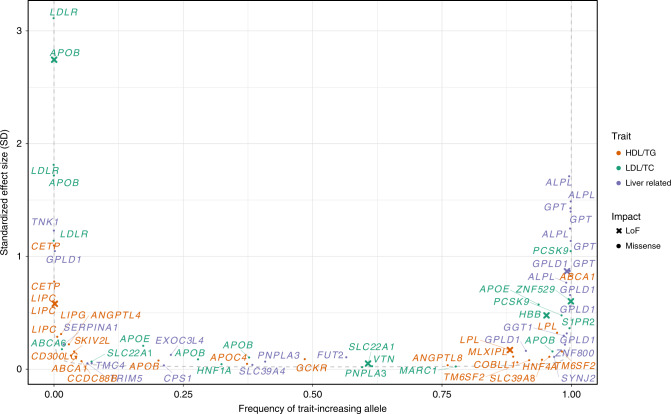
Protein-altering variants with effect on lipid and liver-related blood traits. Smile plot comparing the frequency of the blood-trait increasing allele with the allele’s effect size for protein-altering variants significantly (*P* < 5 × 10^−8^) associated with a lipid (HDL-C, LDL-C, TG, TC) or liver (ALT, ALP, AST, GGT) trait. The most significant trait is shown for variants with significant association for multiple traits. Color indicates the trait category for which the variant is significant, with loss-of-function variants shown as x. Power curve (dashed line) denotes estimated 90% power in the meta-analysis with a sample size of *N* = 210,000 at alpha = 5 × 10^−8^.

Combining all discovery strategies, we identified a total of 674 unique independent variants within 287 loci associated with at least one of the 9 quantitative liver-related blood traits. Of the 287 loci, 92 have not previously been associated with the trait of interest (Supplementary Fig. 4). We identified genome-wide significant associations with at least one trait at 76 presumed causal protein-altering variants, of which 9 result in loss-of-function (LoF) of a specific protein – 3 frameshift indels and 6 premature stop codons (Table 1 and Fig. 1). Eleven of the 76 protein-altering variants, including 1 LoF variant, do not fall within a previously reported locus with respect to the trait of interest.

### Loss-of-function variants with impact on liver-related blood traits

After combining results across all samples and discovery strategies, we were particularly interested in 9 variants annotated to result in LoF of a gene. This included the not previously reported association between *ZNF529*:p.K405X and decreased LDL-C, which we identified in Norwegian samples via sequencing and custom content genotyping (Table 1). We observed 4 additional LoF variants also resulting in substantially decreased LDL-C (3 nonsense variants in *APOB*, and a common frameshift indel in *SLC22A1*; Table 1). The 4 remaining LoF variants are associated with other blood lipid traits (*LPL*:p.S474X with TG, *HBB*:p.Q40X with TC, and *LIPC*:p.G247Afs*11 with HDL-C) or ALP (*GPLD1*:p.V815Sfs*46). The previously reported *LPL*:p.S474X (also known as p.S447X)^17,18^ is, in contrast to other stop-gain variants in the lipoprotein lipase (*LPL*) gene, known to result in gain-of-function of *LPL*^19^. This explains the association with decreased TG and points to LPL activation as a potential mechanism of CVD risk reduction.

**Fig. 2:**
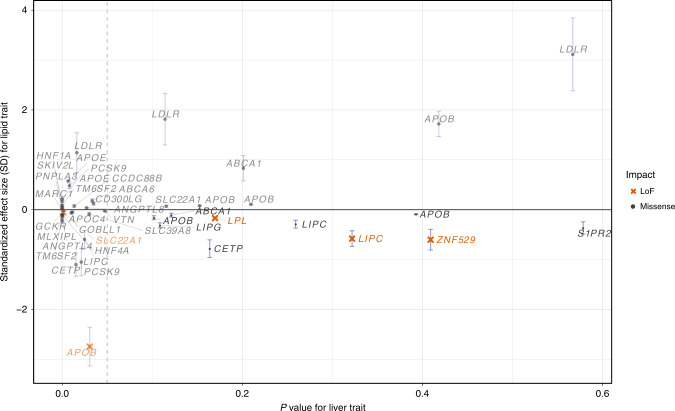
Prioritizing drug targets based on lipid effect and liver enzyme associations. For any variant significantly (*P* < 5 × 10^−8^) associated with a lipid trait (HDL-C, LDL-C, TG, TC), the maximum effect size in terms of the allele associated with good lipid health (e.g., lowered LDL-C, increased HDL-C, lowered TG, and lowered TC) is compared to the minimum *p* value for association with liver trait (ALT, ALP, AST, GGT). Vertical whiskers represent 95% confidence intervals of the effect size. Nominal *P* value of 0.05 (vertical dashed line) is indicated to highlight variants in the bottom right quadrant which lack significance for association with liver traits. These variants are better drug target candidates given estimated favorable lipid-effects on health and absence of association with potentially unfavorable liver traits.

Of the 9 predicted LoF variants, the 4 within *LPL*, *LIPC*, and *ZNF529* were not even nominally significantly associated (*P* > 0.05) with ALT, AST, ALP or GGT (Fig. 2). Although we observed two very rare non-coding variants in proximity to *ZNF529* that are associated with increased ALT (Supplementary Data 2-3 and Supplementary Fig. 5), these variants are completely independent (*r*^2^ < 0.01) of *ZNF529*:p.K405X. Altogether, association results for liver enzymes and blood lipids indicate that hemizygous loss-of-function alleles in *LIPC* and *ZNF529* and gain-of-function alleles in *LPL* do not cause liver damage, prioritizing these genes as potential drug targets to reduce blood lipids and CVD without liver-damaging side effects.

### Functional characterization of ZNF529:p.K405X

We expect that protein-altering variants that are the most strongly associated variants in a region represent functional variants that pinpoint biologically relevant genes and potential drug targets. We also sought not previously described genes that decreased cardiovascular risk factors (such as LDL-C) without increasing risk of liver disease or impact liver enzymes. Thus, we focused on the not previously reported association between *ZNF529*:p.K405X and LDL-C (beta −0.6, *P* = 1.3 × 10^−8^) since this variant is neither associated with liver enzymes (*P* = 0.4−0.9 for all 4 liver enzyme traits, *N* range 21,530–48,569) nor non-fasting blood glucose (*P* = 0.93, *N* = 54,093 individuals) in HUNT.

**Fig. 3:**
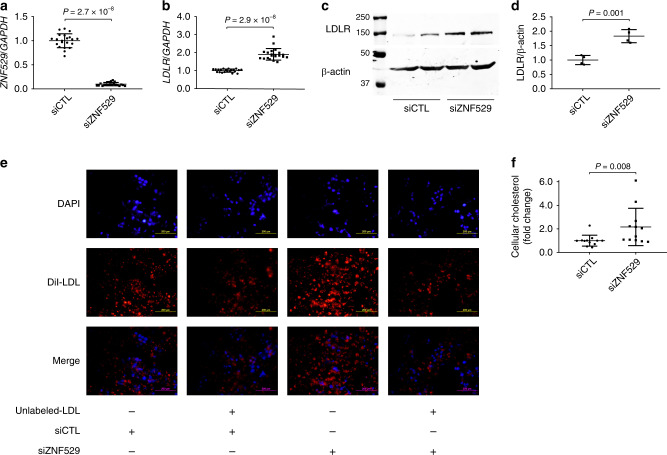
ZNF529 silencing induces LDLR expression and LDL uptake. **a** Efficient silencing of ZNF529 in HepG2 cells via siRNA as shown by qPCR using GAPDH as reference (*N* = 21 biologically independent samples). **b** ZNF529 silencing in HepG2 cells induces LDLR mRNA as shown by qPCR using GAPDH as reference (*N* = 21 biologically independent samples), and (**c** and **d**) LDLR protein as shown by western blot using β-actin as loading control (*N* = 4 biologically independent samples). **e** ZNF529 silencing in HepG2 cells increases LDL uptake as evidenced by enhanced fluorescence of DiI-LDL (10 µg/ml, *N* = 9 biologically independent samples) which is inhibited in cells preloaded with 25-fold excess amounts of unlabeled-LDL (250 µg/ml, *N* = 3 biologically independent samples, scale bars = 200 µm), and (**f**) leads to increased intracellular cholesterol (*N* = 12 biologically independent samples). Values are presented as mean ± SD (vertical whiskers) showing all points and *P* values (two-tailed). Mann–Whitney *U* test was used for **a**, **b** and **f**. Student *t* test was used for **d**. Source data are provided as a Source Data file.

Zinc finger 529 (ZNF529) does not have a homolog in rodents. To experimentally assess the consequence of ZNF529 LoF on cholesterol metabolism, we transiently knocked-down *ZNF529* in human hepatoma HepG2 cells using siRNA (90.1% reduction, *P* = 2.7 × 10^−8^, Fig. 3a, Supplementary Data 6) and conducted an unbiased analysis of gene expression using RNA sequencing. Principal component analysis revealed a distinct gene expression pattern in HepG2 cells following ZNF529 knockdown with a total of 476 differentially expressed genes identified (Supplementary Fig. 6), including a significant upregulation of the LDL receptor (LDLR, FDR = 7.8 × 10^−7^) (Supplementary Data 7). Pathway analysis revealed enrichment of general metabolism pathways, drug metabolism pathways, and lipid-related pathways (statin pathway, plasma lipoprotein remodeling and plasma lipoprotein assembly, remodeling, and clearance, *P* = 6.5 × 10^−4^, *P* = 2.2 × 10^−2^, and *P* = 3.9 × 10^−2^, respectively) (Supplementary Data 8). We confirmed the upregulation of LDLR mRNA by qPCR (90.6% increase, *P* = 2.9 × 10^−8^, Fig. 3b, Supplementary Data 9) and protein by western blot (83.0% increase, *P* = 0.001, Fig. 3c, d, Supplementary Data 10).

We used 1,1’-dioctadecyl- 3,3,3’,3’-tetramethylindocarbocyanine perchlorate (DiI)-labeled LDL to assess the effects of ZNF529 LoF on LDL uptake in HepG2 cells. First, we confirmed that DiI-LDL is taken-up by the cells in a dose-dependent manner, resulting in saturated uptake at 25 μg/ml (Supplementary Fig. 7). Next, we assessed the specificity of the binding in competition experiments between DiI-labeled and unlabeled LDL. Pretreatment of cells with 25-fold excess amounts of unlabeled LDL inhibited the uptake of DiI-LDL (Fig. 3e, Supplementary Fig. 8). After confirming dose-dependent saturation and specificity, we evaluated the effects of ZNF529 silencing on LDL uptake. We found that ZNF529 silencing resulted in a marked increase in DiI-LDL uptake by HepG2 cells which was suppressed in the presence of excess amounts of unlabeled LDL (Fig. 3e). Additionally, we noted a 2.2-fold increase in intracellular cholesterol following ZNF529 knockdown (*P* = 0.008, Fig. 3f, Supplementary Data 11).

Altogether, these findings suggest that *ZNF529* is a regulator of plasma LDL-C via upregulation of hepatic LDLR and enhanced LDL uptake.

### Clinical implications of ZNF529:p.K405X

Individuals heterozygous for *ZNF529*:p.K405X (*N* = 109, minor allele frequency of 0.1%) had a mean LDL-C level of 2.58 mmol/L (99.8 mg/dL) vs. 3.44 mmol/L (133.0 mg/dL) in non-carriers. This reduction in LDL-C of 25% in heterozygous carriers is in the range of what is seen for treatment with 40 mg of statin^20^, and usually corresponds to a relative risk reduction of major cardiovascular events by 20–25%^2^. We only observed one homozygous female carrier. Despite being obese and hypertensive, she was alive at age >90 years and had no diagnosis of cardiovascular disease, liver disease, or diabetes, and had an LDL-C level slightly below average for her age group (3.45 mmol/L [133.4 mg/dL] vs. mean 3.8 mmol/L [147.0 mg/dL] for women >90 years old). This one individual with a natural absence of both copies of *ZNF529* suggests that homozygous knockout of this gene is compatible with survival. We sought to replicate *ZNF529:p.K405X* outside the HUNT Study, but found that the allele count was too low for meaningful association analyses (e.g., 1 copy in 26,638 alleles in the Michigan Genomics Initiative [MGI] and 1 copy in 125,568 alleles in TOPMed). The existence of *ZNF529:p.K405X* in HUNT was confirmed by Sanger sequencing of the homozygous sample and 9 heterozygous samples (100% match between genotyping and Sanger sequencing) (Supplementary Fig. 9).

### Highlight of protein-altering variants with high effect size

We highlight 17 protein-altering variants with an impact >1 standard deviation on the trait (Fig. 1 and Supplementary Data 12). For lipids, protein-altering variants in *APOB, LDLR*, and *PCSK9* that impact LDL-C, and in *CETP* that impact HDL-C, are well known^17^. However, for liver enzyme traits, *TNK1*:p.G574V is a new finding to complement genes previously known to impact or encode liver enzymes including *ALPL* (with ALP)^21^, *GPLD1* (with ALP), and *GPT* (with ALT)^22^. This rare *TNK1* variant, present in 46 individuals (1 in 814 individuals), was first identified in Norwegian sequenced samples, then genotyped using the custom array, and observed to have a large impact on ALP (beta = 1.2, *P* = 1.7 × 10^−14^).

### Gene-based burden tests

Gene-based burden results are, in contrast to single variant tests, independent of nearby signals and may point to the functional gene in a region. To identify genes functionally involved in the 9 liver-related blood traits, we performed gene-based burden tests using SKAT-O as implemented in SAIGE-GENE^23^. We included all protein-altering variants with frequency below 0.5% in the HUNT dataset. Although we found 33 unique genes to be significantly associated (*P* < 2 × 10^−7^) with at least one of 9 liver traits (Supplementary Data 13), in only two cases was the gene-based evidence for association substantially stronger than the strongest single variant. This comprised 10 variants in the gene *GPT* associated with ALT (*P* = 2.35 × 10^−60^ vs. *P* = 6.43 × 10^−25^ for rs147998249) and 6 variants in the gene *ALPL* associated with ALP (*P* = 2.9 × 10^−239^ vs. *P* = 3.99 × 10^−67^ for rs138587317). These data suggest there are multiple, functional coding rare variants in each of these two genes. The gene-based burden test also identified well-known genes such as *CETP* and *ABCA1* associated with HDL-C; *PCSK9, LDLR* and *APOB* associated with LDL-C; the *CRP* gene associated with CRP; and *GPLD1* associated with ALP (Supplementary Data 13). Additionally, burden tests for the 3 LoF *APOB* variants indicated no association with liver enzymes for heterozygous LoF carriers (Supplementary Data 14), highlighting *APOB* as another potentially valuable pharmaceutical target for blood lipid lowering.

### Cross-trait analyses for evaluating potential consequences of gene targeting

**Fig. 4:**
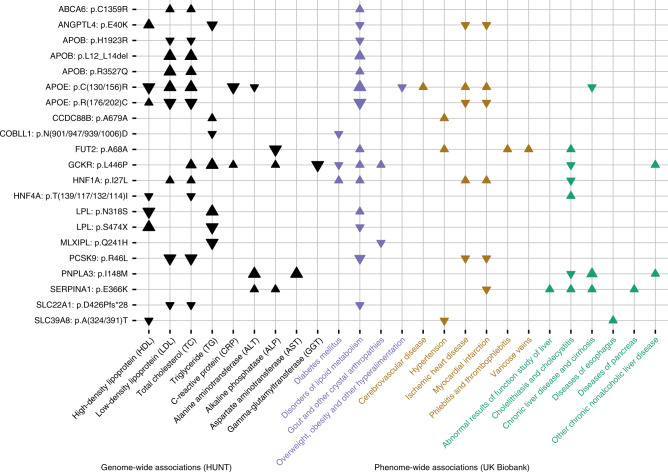
Phenome-wide association study in UK Biobank (*N* = 408,961 participants) based on presumed causal protein-altering variants with impact on liver-related blood traits in The HUNT Study (*N* = 69,479). The figure displays phenome-wide statistically significant (*P* < 3.5 × 10^−5^) associations between selected protein-altering variants (*n* = 21) with impact on one or more of the 9 liver-related blood traits and selected cardiovascular, liver, and metabolic phenotypes derived from ICD codes in UK Biobank. Arrows denote the direction of effect for the minor allele. Larger arrows signify more significant associations. Statistically insignificant associations are not displayed. Please see Supplementary Fig. 10 and Supplementary Data 15 for the full phenome-scan across all traits and variants available for testing in UKB (*n* = 24). The ZNF529 LoF variant could not be imputed into the UK Biobank.

To expand our understanding of the 76 presumed causal protein-altering variants, to investigate their impact on disease, and to evaluate potential consequences of targeting the implicated gene or its gene product, we imputed the TOPMed reference panel into the UK Biobank and performed a phenome-wide association study (PheWAS) across 1342 ICD code-defined disease groups^24,25^, in 408,961 people of white British ancestry. Sixty-four of the 76 protein-altering variants could be imputed sufficiently well (*R*^2^ > 0.3). Of 64 variants assessed, we found that 24 variants are associated with one or more diseases at a phenome-wide significance level (*P* < 3.5 × 10^−5^) (Fig. 4, Supplementary Fig. 10, and Supplementary Data 15).

To identify potentially useful pharmaceutical strategies that may reduce blood lipid levels and risk of coronary artery disease (CAD) and type 2 diabetes (T2D) without increasing the risk of fatty liver disease, we attempted to identify variants that decreased LDL-C or TG and decreased the risk of cardio-metabolic disease, but were not associated with changes in liver enzyme levels (*P* > 0.05, Figs. 2, 4, Supplementary Fig. 10 and Supplementary Data 12–15), suggesting that liver function was not altered. The 2 variants with this pattern of association include (i) *COBLL1*:p.N497D which is associated with decreased TG levels and decreased risk of T2D in both HUNT and UK Biobank and of liver disease in HUNT and (ii) *LPL*:p.S474X which is associated with decreased risk of CAD, T2D, and hypertension. HDL-C-associated *ANGPTL4* p.E40K appears to decrease risk of T2D, CAD, and hypertension but is also associated with an increased risk of ankylosing spondylitis (Supplementary Fig. 10 and Supplementary Data 15), hence a potential complication of targeting this gene.

From the PheWAS, we observed other interesting associations. For example, we found that the variant *SERPINA1*:p.E366K, which is known to cause alpha-1-antitrypsin deficiency, often complicated by severe liver and pulmonary disease^26^, is also associated with a decreased risk of myocardial infarction. This finding supports clinical evidence that individuals with alpha-1-antitrypsin deficiency may be protected against coronary artery disease^27^. The association, however, also suggests caution in ongoing efforts to treat acute myocardial infarction with exogenous administration of alpha-1-antitrypsin^28,29^, because the genetic association suggest the opposite effect – an increased risk of myocardial infarction. Another interesting finding is the low-frequency *S1PR2* variant p.Y257C (chr19:10224136T>C, MAF = 0.003) which we found to be associated with decreased LDL-C (effect of -0.37 SD, *P* = 6 × 10^−9^) and a decreased risk of coronary artery disease, including myocardial infarction (odds ratio 0.45, *P* = 0.0005), without being associated with liver enzyme traits (Supplementary Data 12).

Further studies are obviously warranted to uncover the biological mechanisms underlying the associations described here, however, each of them could help inform clinical implications of targeting the underlying gene or gene product.

## Discussion

By using complementary approaches for genomic association discovery: sequencing, imputation, array-based genotyping, stepwise conditional analyses and trans-ancestry meta-analyses, we identified >650 independent genomic variants associated with quantitative liver-related disease precursors. This included 76 protein-altering variants that we assume to be causally related to one or more of the traits. By broadly considering associations between these protein-altering variants, quantitative traits, and disease endpoints, we prioritize several genes as potential pharmaceutical targets for preventing or treating CVD.

The newly uncovered association and in vitro studies indicate that *ZNF529* LoF is associated with lower plasma LDL-C, which could be explained by induction of LDLR in hepatic cells and increased LDL uptake. While these findings indicate a therapeutic potential for lowering plasma LDL-C by ZNF529 inhibition, further studies are warranted to elucidate the mechanisms by which ZNF529 regulates LDLR and LDL uptake in the liver. Considering that ZNF529 does not have a homolog in rodents, the use of animal models for such studies is limited.

Another interesting finding to highlight is the presumed causal low-frequency protein-altering variant *S1PR2*:p.Y257C that we found to be associated with decreased LDL-C and a >50% reduction in the risk of myocardial infarction, without being associated with altered liver function or non-fasting blood glucose. The variant was identified via low-pass sequencing and custom content genotyping in HUNT. *S1PR2* encodes sphingosine-1-phosphate receptor 2, which seems to play a critical role in the endothelial inflammatory response^30^. Several animal models have already indicated that inhibition of *S1PR2* could be a valuable pharmaceutical target for vascular recovery in coronary artery disease and stroke^30–32^. The associations that we report here represent the first direct human data supporting that S1PR2 might play an important role in ischemic heart disease.

Taken together, we demonstrate that identifying rare protein-altering variants and careful consideration of multiple phenotypes in well-powered studies may point to promising pharmaceutical drug targets. We used a variety of approaches to identify rare protein-altering variants, and we found that if exome sequencing is prohibitively expensive, sequencing a subset of samples followed up with a custom genotyping array can be a viable strategy to identify impactful rare variants.

## Methods

### The HUNT Study

The Trøndelag Health Study (HUNT) is a population-based health survey conducted in the county of Trøndelag, Norway, since 1984^9^. Participation in the HUNT Study is based on informed consent and the study has been approved by the Data Inspectorate and the Regional Ethics Committee for Medical Research in Norway (REK: 2014/144). We included a total of 69,479 individuals with values for at least one of the traits examined (ALT, ALP, AST, CRP, GGT, HDL-C, LDL-C, TC and TG). Genotyping was performed using the Illumina Human CoreExome v1.1 array with 70,000 additional custom content beads^33,34^. Variants were selected for genotyping if they were: protein-altering (*n* = 13,618); modestly associated with lipids in HUNT but not tested in large consortia (*n* = 960); identified as causing familial hypercholesterolemia in Norwegian patients (*n* = 110); or predicted to result in a loss-of-function of one of the 56 ACMG genes (*n* = 27,144, Supplementary Data 16). Additionally, we selected missense variants with 2 or more copies (*n* = 8720) and nonsense variants with 1 or more copy (*n* = 756) identified from low-pass whole-genome sequencing of 2202 HUNT samples. Please see Supplementary Data 17 for a summary of selected custom content variants.

Imputation was performed from 60,039 TOPMed reference genomes using Minimac3 and variants with imputation quality >0.3 were retained. To account for relatedness within the sample, we performed association testing using the linear mixed model with genetic relationship matrix as implemented in SAIGE [https://github.com/weizhouUMICH/SAIGE]^12^. Birth year, sex and PC1-4 were included as covariates. Conditional analysis was performed with the same analysis tools and command line options as the association analysis by adding the lead-SNP(s) in a step-wise manner as covariate(s) into the SAIGE step1 parameter estimation until the variant with smallest *P* value in the locus was >5 × 10^−8^.

### Biobank Japan

Biobank Japan (BBJ) is a multi-institutional hospital-based registry of ~200,000 individuals from 66 Japanese hospitals collected from 2003 to 2007. ﻿Written informed consent was obtained from all participants, as approved by the ethics committees of RIKEN Center for Integrative Medical Sciences and the Institute of Medical Sciences, the University of Tokyo. Genotype, imputation, and QC were performed as described previously^14^. Briefly, samples were genotyped with Illumina HumanOmniExpressExome or a combination of the Illumina HumnOmniExpress and HumanExome BeadChips and imputed using 1000 Genomes Project Phase 1 version 3 East Asian reference haplotypes. Publicly available summary statistics from linear regression assuming an additive model for quantitative measures of ALP, ALT, AST, CRP, GGT, HDL-C, LDL-C, TC, and TG were used. Quantitative traits were adjusted for age, sex, top 10 PCs of genetic ancestry, and disease status for 47 target diseases. Sample sizes for traits ranged from 70,567 to 134,182^14^.

### SardiNIA

6602 individuals from four villages in the Lanusei valley on Sardinia (>60% of the adult population) were genotyped on four different Illumina Infinium arrays: OmniExpress, Cardio-Metabochip^35^, Immunochip^36^, and Exome Chip. Low-depth (~4x coverage) whole-genome sequencing on 3839 individuals was performed, of which 2340 were also genotyped. Imputation of 1.1 million indels and 24.1 million biallelic single nucleotide variants was performed using Minimac3^37^ and markers with imputation quality >0.3 (or >0.6 if MAF<1%) were retained. Samples, genotyping, sequencing and variant calling have been previously described^38^.

Liver traits (ALT, AST, CRP, GGT, HDL-C, TC, and TG) from the first visit were used and LDL-C was computed using the Friedewald Equation^13^. Association analyses were performed for liver traits assessed in 5570–5942 individuals (median *N* = 5917) using age, age^2^ and sex-adjusted inverse-normalized residuals of the outcomes as input to the Efficient Mixed Model Association eXpedited (EMMAX)^39^ single variant test (i.e., a linear model with a kinship matrix) as implemented in EPACTS [https://github.com/statgen/EPACTS]. Genomic control correction was not applied as the lambda values were not inflated (range 0.97–1.02, Supplementary Data 18). The study, including the protocols for subject recruitment and assessment, the informed consent for participants (or consent from their legally authorized representative for those 14–17y); and the overall analysis plan was reviewed and approved by institutional review boards for the Istituto di Ricerca Genetica e Biomedica (IRGB; Cagliari, Italy), for the MedStar Research Institute (responsible for intramural research at the National Institutes of Aging, Baltimore, Maryland, United States), and for the University of Michigan (Ann Arbor, Michigan, United States).

### Meta-analyses

As the summary statistics from SardiNIA and Biobank Japan were in Human Genome Build hg19, the positions were mapped to Human Genome Build hg38 using liftOver [https://genome.ucsc.edu/cgi-bin/hgLiftOver]. The genomic control corrected summary statistics from the contributing cohorts were combined with METAL^40^ using inverse variance weighted meta-analysis. Meta-analysis included SardiNIA, Biobank Japan, and HUNT for all traits, with the exception of ALP which was only available from HUNT and Biobank Japan.

### Definition of independent loci

Independent loci were defined as genetic markers >1 Mb apart in physical distance with at least one genetic variant associated with the trait of interest at a genome-wide significance threshold of *P* < 5 × 10^−8^. Loci borders were defined as the highest and lowest genomic positions within the locus reaching genome-wide significance plus an additional 1 Mb on either side.

### Novelty of identified genomic loci

A locus was classified as known if a variant previously published to be associated with the trait of interest fell with 1Mb of the locus lead variant that we identified. Otherwise, a locus was classified as novel. Previously published variants were extracted from papers and the GWAS catalog [https://www.ebi.ac.uk/gwas/] at the time of analyses.

### PheWAS in UK Biobank

Association results for 1342 trait groups (PheCodes)^24^ in UK Biobank were generated using SAIGE^12^. Phenotypes were grouped by combining ICD-9 and ICD-10 codes of closely related traits following previously published methods^25^. Analyses were performed on the white British subset of UK Biobank after imputation with the TOPMed reference panel. Sex, birth year, and 4 principal components were included as covariates. Significance was determined based on Bonferroni correction for the number of traits tested (*P* < 3.5 × 10^−5^). Participation in the UK Biobank is based on informed consent^41^.

### Gene-based SKAT-O tests

The exome-wide gene-based SKAT-O tests were performed using SAIGE-GENE v36^23^ for all 9 liver traits based on the TOPMed-imputed HUNT data. Missense and stop-gain variants annotated by ANNOVAR^42^ with MAF ≤ 0.005 were included. Conditional analyses were performed to condition on the most significant single variant association signal within 500 kB of the gene. We selected a significance threshold of 2.5 × 10^−6^ accounting for 20,000 genes.

### Replication attempt of ZNF529:p.K405X in MGI

The Michigan Genomics Initiative (MGI) is a repository of electronic medical record and genetic data at Michigan Medicine (N~58,000 participants). MGI participants were enrolled during pre-surgical encounters at Michigan Medicine and provided consent to study genetic and electronic health record data for research. The MGI study was approved by the Institutional Review Board of the University of Michigan Medical School. DNA was extracted from blood samples and participants were genotyped using Illumina Infinium CoreExome-24 bead arrays, which includes the same custom content as the HUNT Study. Genotype data were imputed to the Haplotype Reference Consortium using the Michigan Imputation Server, providing 17 million imputed variants after standard quality control and filtering. Only European individuals were used for analysis. We attempted to replicate the association with *ZNF529:p.K405X* in 13,319 MGI participants with LDL-C measurements, however, only 1 participant was heterozygous for *ZNF529:p.K405X* so the power to detect association was near zero. In contrast, we identified 110 heterozygous individuals in the HUNT discovery study.

### HepG2 cells

The HepG2 human hepatoma cell line was obtained from the American Type Culture Collection (ATCC) and cultured at 37 °C and 5% CO_2_ in Dulbecco’s Modified Eagle Medium (DMEM, Gibco) supplemented with 10% fetal bovine serum (FBS, Sigma-Aldrich) and 1% Penicillin-Streptomycin (Pen-Strep, Gibco).

### ZNF529 gene silencing using small interfering RNA (siRNA)

siRNA targeting zinc finger protein 529 (siZNF529: GGCUUUUGGAGUAUGUAGAtt) and non-targeting siRNA control (siCTL) were obtained from Ambion (siRNA IDs s33654 and AM4611, respectively). HepG2 cells were transfected with 20 nM of siZNF529 or siCTL using Lipofectamine RNAiMAX (Invitrogen) in Opti-MEM reduced-serum medium (Gibco) in accordance with the manufacturer’s protocol^43^. Cellular lipid or protein extraction, RNA isolation or LDL uptake assays were conducted 48 h post transfection.

### RNA sequencing

Total RNA was purified from HepG2 cells using the QIAGEN’s RNeasy kit (QIAGEN). Library preparation and sequencing were performed by the University of Michigan DNA Sequencing Core. RNA was assessed for quality using the TapeStation (Agilent, Santa Clara, CA). All samples had RNA integrity numbers (RINs) >8.5. Samples were prepared using the NEBNext Ultra II Directional RNA Library Prep Kit for Illumina (NEB, E7760L) with Poly(A) mRNA Magnetic Isolation Module (NEB, E7490L) and NEBNext Multiplex Oligos for Illumina Unique dual (NEB, E6440L), where 10 ng–1 µg of total RNA were subjected to mRNA polyA purification. The mRNA was then fragmented and copied into first strand cDNA using reverse transcriptase and dUTP mix. Samples underwent end repair and dA-Tailing step followed by ligation of NEBNext adapters. The products were purified and enriched by PCR to create the final cDNA library. Final libraries were checked for quality and quantity by TapeStation (Agilent) and qPCR using Kapa’s library quantification kit for Illumina Sequencing platforms (Kapa Biosystems, KK4835). Libraries were paired-end sequenced on a NovaSeq 6000 Sequencing System (Illumina).

Paired-end reads (101 bp) from RNA sequencing of 4 siCTL and 4 siZNF529 samples were aligned to hg38 reference genome using Tophat2 (v2.0.13 11/5/19 7:29:00 PM)^44^ with default parameters. In each sample, 94.3–95.4% reads could be aligned. All valid alignments were used for downstream analysis. GENCODE release 29 was used to obtain gene boundaries of 19,940 protein coding genes. We used Samtools (v1.9)^45^ and bedtools (v2.22.0)^46^ coverage function to count number of reads aligned in each genic bin. Genes with >3 counts per million (CPM) in 8 samples were used for further analysis. EdgeR^47^ library in R was used to identify differentially expressed genes (Supplementary Data 7). We used glmFit followed by glmTreat to identify significant change in expression with FDR < 0.05. We used ConsensusPathDB^48^ to identify enriched pathways from list of differentially expressed genes (Supplementary Data 8).

### RNA isolation, RT-PCR and qPCR

Total RNA was purified from HepG2 cells using the QIAGEN’s RNeasy kit (QIAGEN). cDNA was synthesized using SuperScript III (Invitrogen), and qPCR was performed using SYBR green reagents (Bio-Rad). Gene expression is presented as fold-change compared with RNA isolated from control cells by the comparative CT (2^−ΔΔCT^) method using GAPDH as the reference gene^49,50^. Primer pairs used for qPCR were obtained from Integrated DNA Technologies and are available in Supplementary Data 19.

### Protein extraction and western blot

Cells were lysed in radioimmunoprecipitation assay lysis buffer (RIPA buffer, Thermo Scientific) supplemented with a protease inhibitor cocktail (Roche Applied Science). Proteins were resolved in 8% sodium dodecyl sulfate polyacrylamide gel electrophoresis (SDS-PAGE) and transferred to nitrocellulose membranes (Bio-Rad). The membranes were blocked for 1 h at room temperature in tris-buffered saline-Tween 20 (TBST) containing 5% fat-free milk and incubated with primary antibody at 4 °C overnight. The following primary antibodies were used: rabbit monoclonal anti-LDLR antibody (Abcam, ab52818, working dilution 1:1000) and mouse monoclonal anti-β-actin antibody (Cell signaling, 8H10D10, working dilution 1:2000). After TBST washing, membranes were incubated with secondary antibodies (LI-COR Biotechnology, donkey anti-rabbit IRDye 926-32213 and donkey anti-mouse IRDye 926-68072, working dilution 1:10000) for 1 h at room temperature. After TBST washing, bands were visualized and quantified using an Odyssey Infrared Imaging System (LI-COR Biosciences, version 2.1)^49^.

### DiI-LDL uptake assay

1,1’-dioctadecyl- 3,3,3’,3’-tetramethylindocarbocyanine perchlorate-low-density lipoprotein (DiI-LDL Alfa Aesar, J65330 or Kalen Biomedical, 770230-9) was used to evaluate the cellular uptake of LDL^51^ in accordance with the manufacturer’s instructions. Briefly, 48 h following siRNA transfection, HepG2 cells were washed with PBS (x2) and changed to serum-free DMEM supplemented with 0.1% bovine serum albumin (BSA, Sigma-Aldrich). The cells were then incubated with DiI-LDL (1–25 μg/ml) for 5 h at 37 °C in the dark. In some experiments, cells were pre-treated for 30 min with 25-fold excess amounts of unlabeled LDL (Alfa Aesar, J65039) to assess the specificity of the binding. Nuclei were stained with 4′,6-diamidino-2-phenylindole (DAPI, Cayman Chemical Company, 14285). After incubation, the cells were washed with PBS (x2) and changed to serum- and probe-free DMEM. Finally, the cells were visualized using a fluorescent microscope (Olympus, IX71). For each experiment two random fields were chosen and photographed in a blinded fashion. DiI-LDL and DAPI images were merged using ImageJ software (v.1.52k, NIH).

### Lipid extraction and cholesterol quantification

The lipids of HepG2 cells were extracted using hexane (≥99%, 32293, Sigma-Aldrich) and isopropanol (≥99.5%, A426-4, Fisher Chemicals) at a 3:2 ratio (*v:v*), and the hexane phase was left to evaporate for 48 h. The remaining cells in the plates were disrupted in 0.1 M NaOH for 24 h and an aliquot was taken for measurement of cellular protein using the Bradford protein assay (Bio-Rad). The content of cellular cholesterol was determined spectrophotometrically using a commercially available kit (Wako Chemicals, 999-02601). Cholesterol data were normalized to cellular protein levels^49,52^.

### Statistical analyses for in vitro studies

Statistical analyses were performed using SPSS 24.0 software (SPSS Inc. IBM). Unless indicated otherwise, values are presented as mean ± SD showing all points. All data were tested for normality and equal variance. If the data passed those tests, Student *t* test was used for comparisons between the two groups. If the data did not pass those tests, nonparametric Mann–Whitney *U* test was used. *P* < 0.05 was considered statistically significant.

### Reporting summary

Further information on research design is available in the Nature Research Reporting Summary linked to this article.

## Supplementary information

Supplementary Information

Peer Review File

Description of Additional Supplementary Files

Supplementary Data

Reporting Summary

## Data Availability

All relevant data supporting the key findings of this study are available within the article and its Supplementary Information or Source data files or from the corresponding author upon reasonable request. The genome-wide summary association statistics are available for download at http://csg.sph.umich.edu/willer/public/hunt-lipids-liver-2020/ or at http://jenger.riken.jp/en/ for data related to Biobank Japan. The raw RNA sequence reads (accession number PRJNA549711) are available for download at https://www.ncbi.nlm.nih.gov/bioproject/PRJNA549711/. Source data are provided with this paper.

## Source data

Source Data
