# Supplementary material for: Loss-of-function genomic variants highlight potential therapeutic targets for cardiovascular disease
Source: Nat Commun. 2020 Dec 18;11:6417. doi: 10.1038/s41467-020-20086-3 (PMC7749177; doi:10.1038/s41467-020-20086-3)
Supplement: Supplementary file 1 — Supplementary Information [file 41467_2020_20086_MOESM1_ESM.pdf]

SUPPLEMENTARY INFORMATION

'Loss-of-function genomic variants highlight potential therapeutic targets for cardiovascular disease' by Nielsen J.B., Rom O. et al., Nature Communications 2020.

SUPPLEMENTARY FIGURES

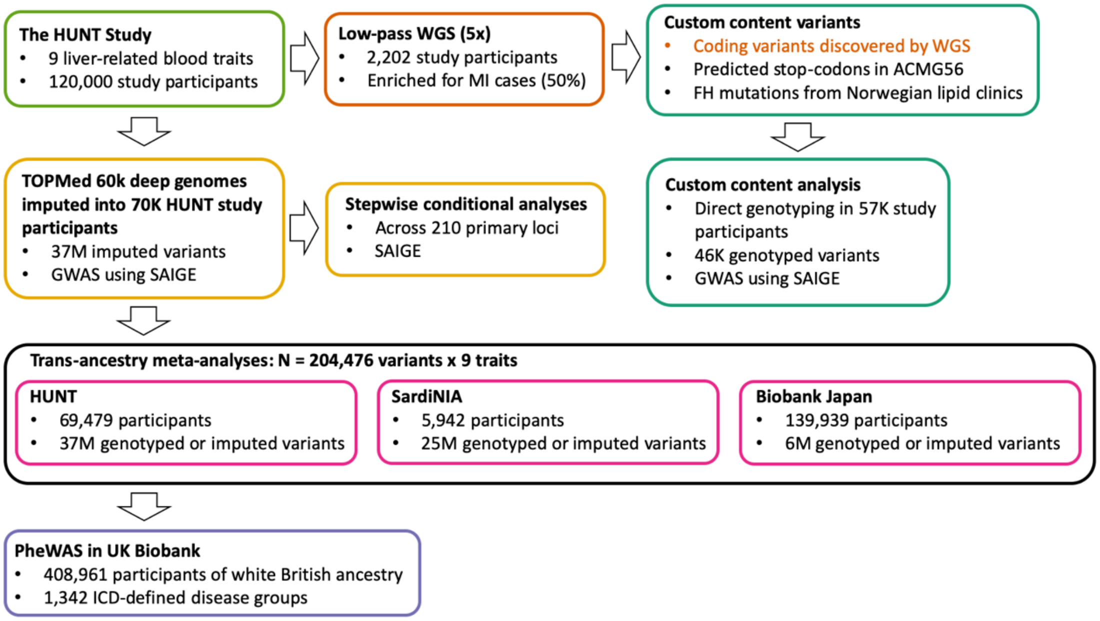

Supplementary figure 1 – Study design.

Approximately 70,000 individuals from the HUNT study have been genotyped and have available biomarker data. Included in the genotyping array for these individuals were custom content variants selected based on variants identified from whole-genome sequencing of Norwegians, known variants associated with lipid phenotypes, and predicted stop codons in ACMG genes. We analyzed the genotyped variants directly for association with 9 liver-related phenotypes, analyzed the TOPMed imputed results, and performed trans-ethnic meta-analysis with two additional cohorts. MI; myocardial infarction, WGS; whole genome sequencing, ICD; international classification of diseases, SAIGE; Scalable and Accurate Implementation of GEneralized mixed model.

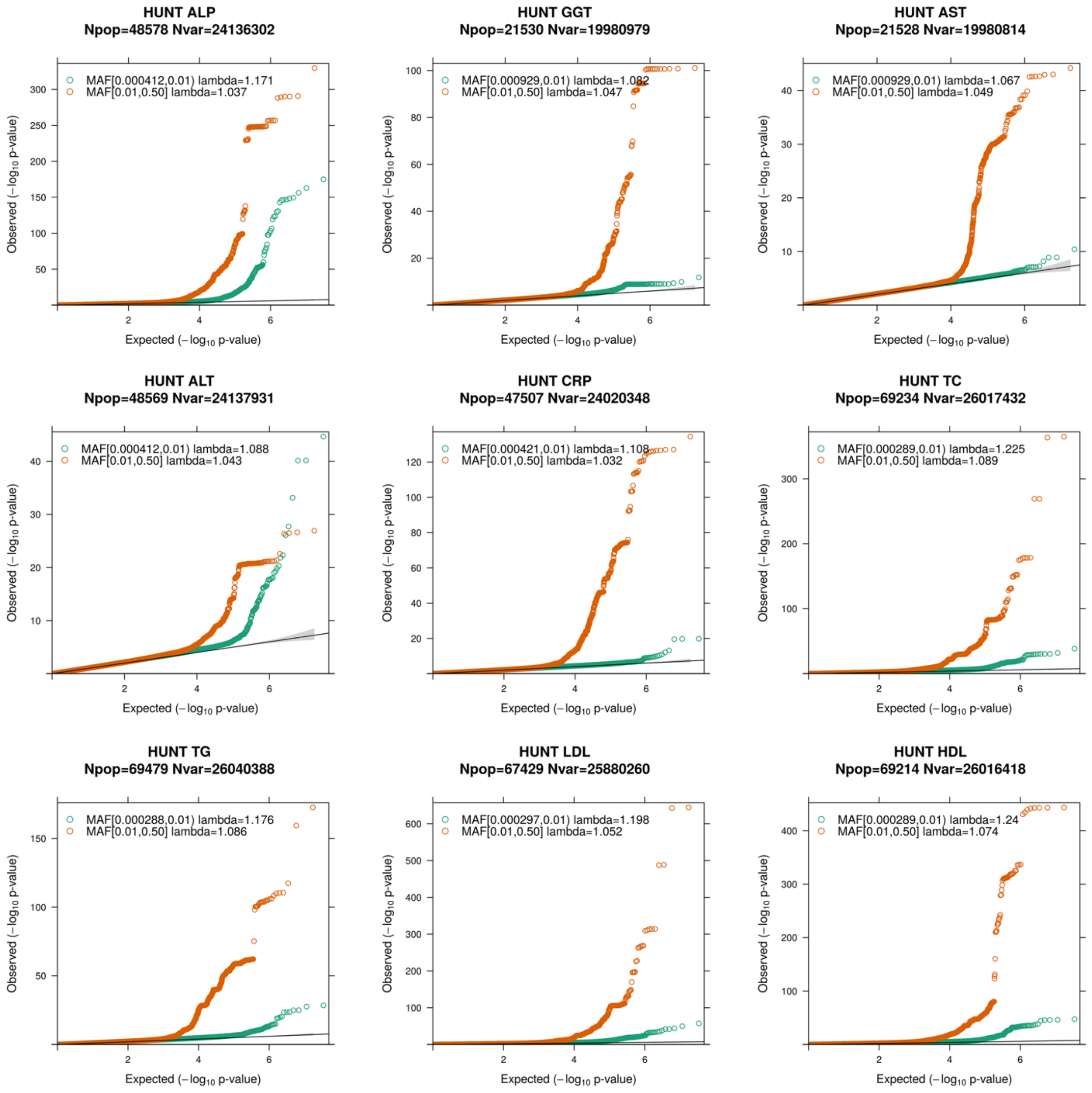

Supplementary figure 2 – QQ plots from HUNT analyses.

Markers are stratified by minor allele frequency below vs. above 0.01. Dots indicate observed P-values (–$\log_{10}$[P-value]) compared with those expected by chance under the null hypothesis (no association). The black line indicates the identity (no association) with corresponding 95% confidence intervals.

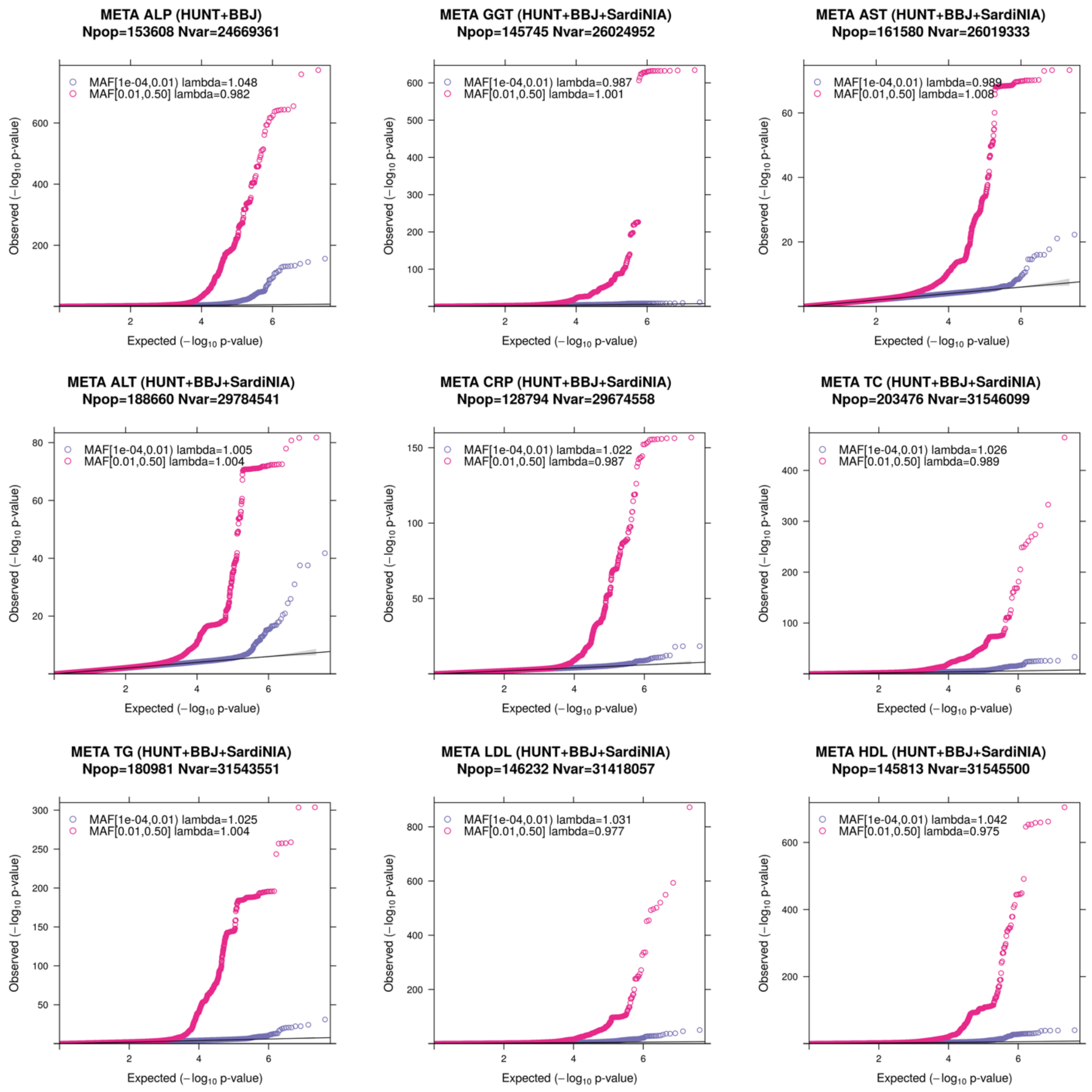

Supplementary figure 3 – QQ plots from meta-analyses.

Markers are stratified by minor allele frequency below vs. above 0.01. Dots indicate observed P-values ($-\log_{10}$[P-value]) compared with those expected by chance under the null hypothesis (no association). The black line indicates the identity (no association) with corresponding 95% confidence intervals.

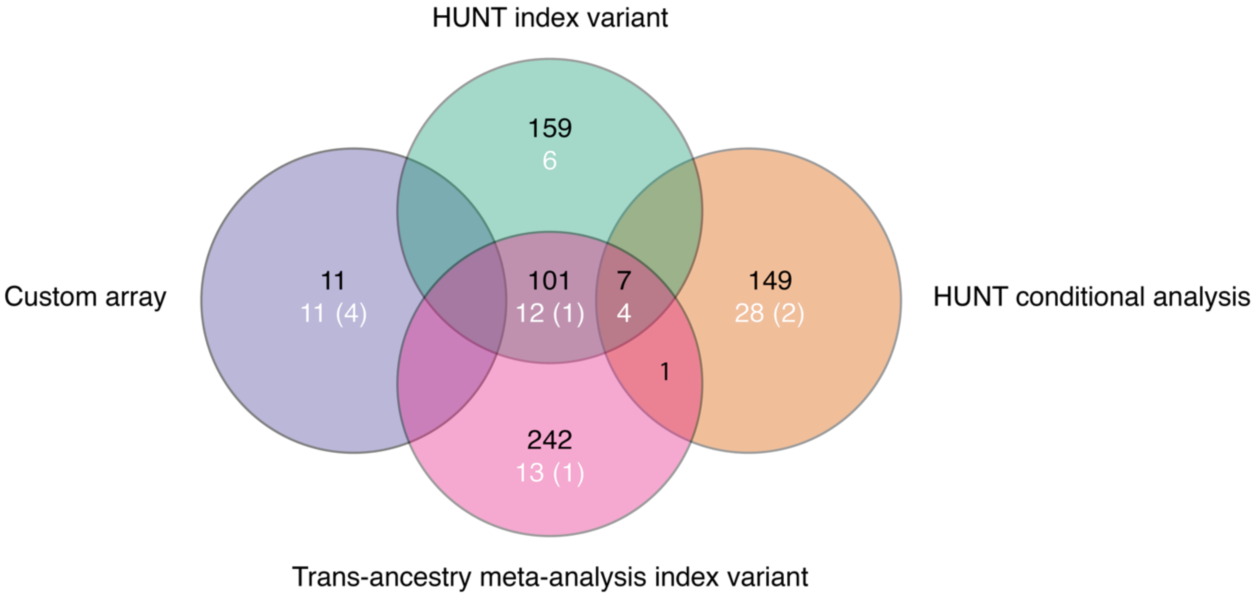

Supplementary figure 4 – Genome-wide significant results generated by each methodology.

The number of index variants that reached genome-wide significance are indicated on the Venn diagram by the four different methodological approaches and their overlap. The black numbers indicate the index variants, white indicates index variants that are coding variants, and the number in brackets indicates index variants that are LoF.

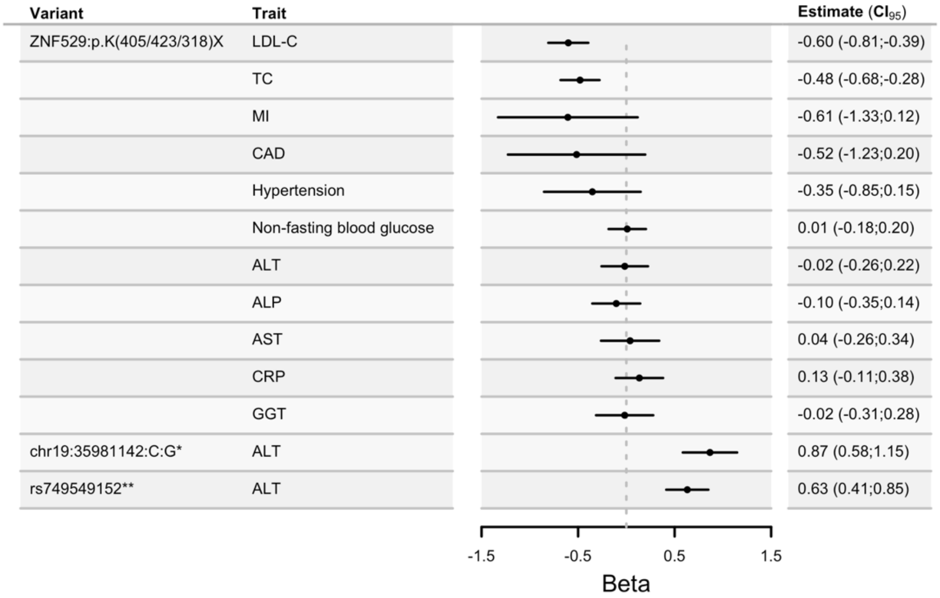

Supplementary figure 5 – Association pattern for variants in proximity to ZNF529 that were associated with one or more traits at a genome-wide significance level ($P<5x10^{-8}$).

*Beta is reported with respect to 1 SD change (LDL-C, TC, ALT, ALP, AST, CRP, GGT) or disease status (MI, CAD, hypertension). *Variant completely independent with p.K(405/423/318)X (r^2 <0.001). **Variant identified through conditional analyses and current association analysis is conditioned on chr19:35981141:C:G. The variant itself is completely independent with p.K(405/423/318)X (r^2 <0.001).*

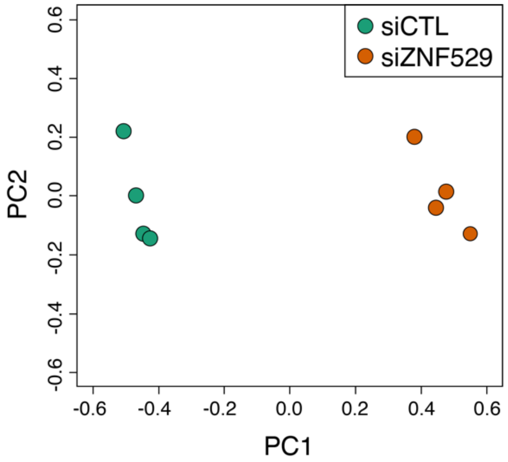

Supplementary figure 6 – A distinct gene expression pattern in HepG2 cells following ZNF529 knockdown.

HepG2 cells were transfected with siRNA targeting ZNF529 (siZNF529, orange dots, N=4) or non-targeting siRNA control (siCTL, green dots, N=4 biologically independent samples). Following 48h, RNA was purified from the cells and used for library preparation and sequencing on a NovaSeq 6000 System. Principal component analysis is shown underlining a distinct gene expression pattern in HepG2 cells transfected with siZNF529 vs. siCTL.

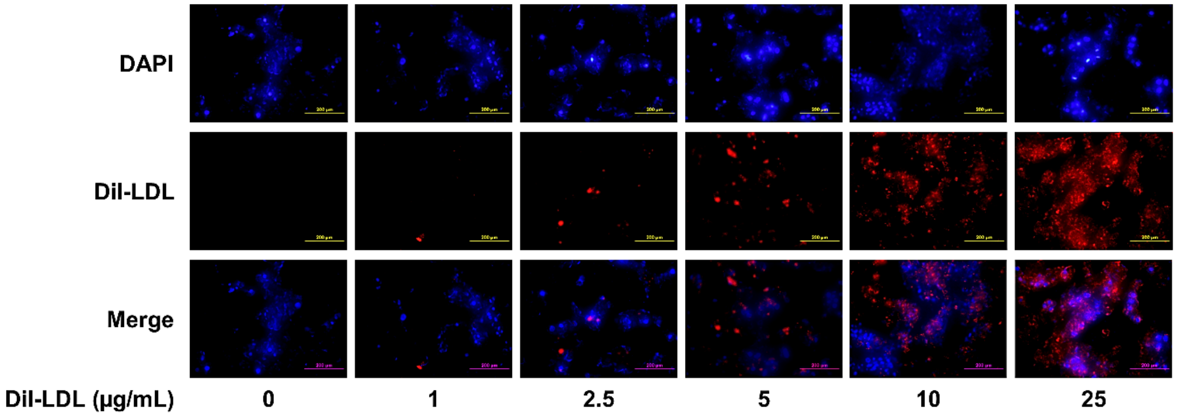

Supplementary figure 7 – Dose-dependent saturation of DiI-LDL uptake.

HepG2 cells were incubated for 5 h with increasing levels of DiI-LDL (0-25 µg/ml) in serum-free DMEM. Nuclei were stained with DAPI 30 min prior to visualization by fluorescent microscopy (N=3 biologically independent samples). DiI-LDL was taken-up by the cells in a dose-dependent manner, resulting in saturated uptake at 25 µg/ml (scale bars=200 µm). Source data are provided as a Source Data file.

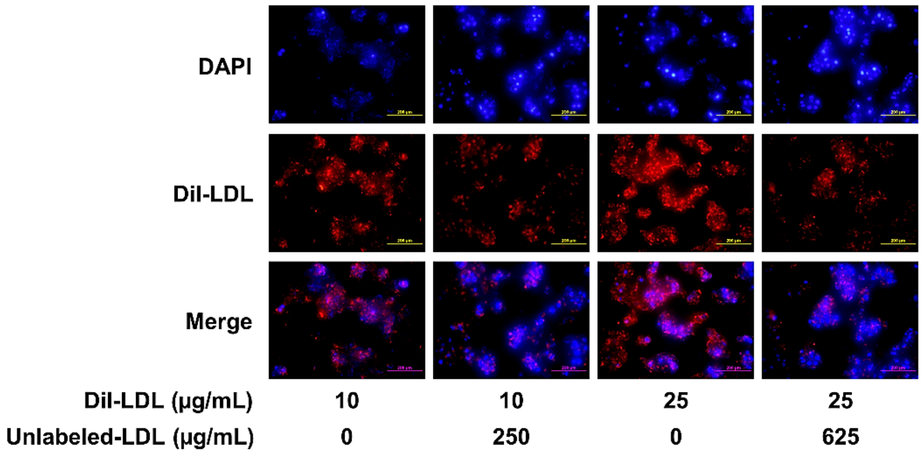

Supplementary figure 8 – Competitive binding between DiI-labeled and unlabeled LDL.

To assess the specificity of the binding, HepG2 cells were incubated for 5 h with DiI-LDL (10 or 25 μg/ml) in serum-free DMEM with or without pretreatment with 25-fold excess amounts of unlabeled LDL (250 or 625 μg/ml). Nuclei were stained with DAPI 30 min prior to visualization by fluorescent microscopy (N=3 biologically independent samples). The uptake of DiI-LDL was inhibited in the presence of 25-fold excess amounts of unlabeled LDL (scale bars=200 μm). Source data are provided as a Source Data file.

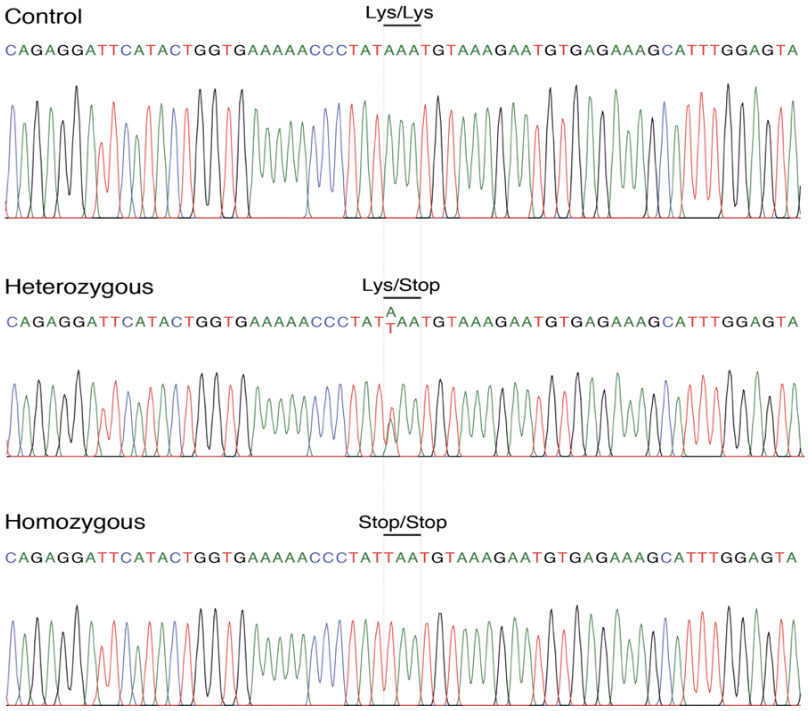

Supplementary figure 9 – Confirmation of ZNF529:p.K(405/423/318)X using Sanger sequencing.

The figure shows the raw chromatograms from one control, one heterozygous carrier, and the only homozygous carrier.

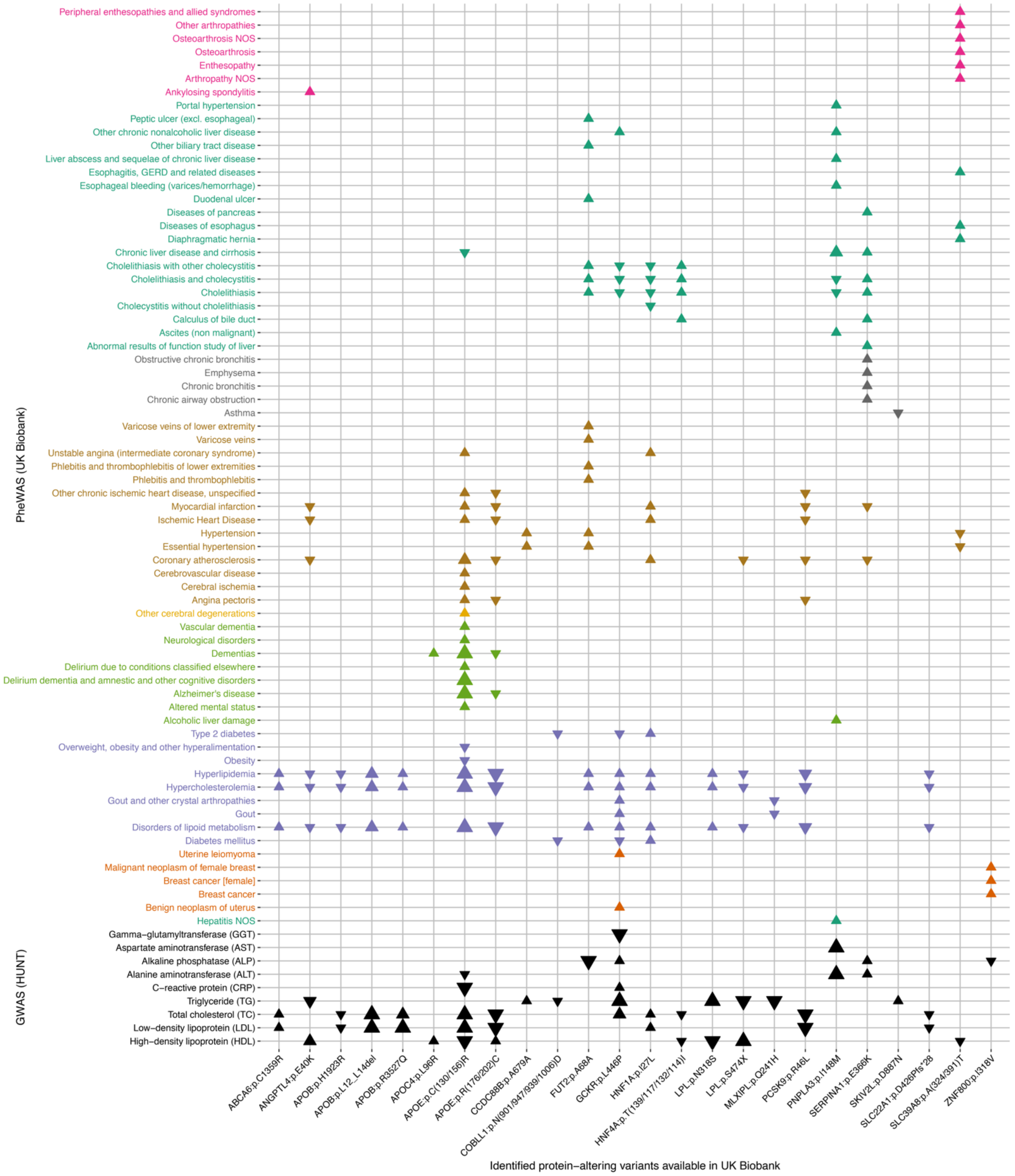

Supplementary figure 10 – UK Biobank PheWAS results for all protein-altering variants.

A total of 24 protein-altering variants were statistically significantly ($P < 3.5x10^{-5}$) associated with one or more ICD code-defined diseases in UK Biobank. Arrows denote the direction of effect for the minor allele. Larger arrows signify more statistically significant associations. Statistically non-significant associations are not shown.
