# Supplementary material for: Loss-of-function genomic variants highlight potential therapeutic targets for cardiovascular disease
Source: Nat Commun. 2020 Dec 18;11:6417. doi: 10.1038/s41467-020-20086-3 (PMC7749177; doi:10.1038/s41467-020-20086-3)
Supplement: Supplementary file 3 — Description of Additional Supplementary Files [file 41467_2020_20086_MOESM3_ESM.pdf]

Description of Additional Supplementary Files

File Name: Supplementary table 1.

Description: Sample sizes for 9 liver traits examined in the HUNT study. A subset of.

File Name: Supplementary table 2.

Description: 271 unique index variants (P < 5x10-8) for one or more traits within 201 loci (representing 329 significant variant-trait combinations).

File Name: Supplementary table 3.

Description: 161 unique variants with P < 5x10-8 identified by conditional analysis of the 201 primary loci.

File Name: Supplementary table 4.

Descriptions: 351 unique variants identified from transethnic meta-analysis (426 variant-trait associations).

File Name: Supplementary table 5.

Description: Protein-altering variants identified from analysis of custom content.

File Name: Supplementary table 6.

Description: ZNF529 mRNA levels following its knockdown in HepG2 cells (Figure 2a).

File Name: Supplementary table 7.

Description: Genes with altered expression pattern following ZNF529 silencing in HepG2 cells.

File Name: Supplementary table 8.

Description: Pathway enrichment analysis using 476 differentially expressed genes.

File Name: Supplementary table 9.

Description: LDLR mRNA levels following ZNF529 silencing (Figure 2b).

File Name: Supplementary table 10.

Description: LDLR protein abundance following ZNF529 silencing (Figure 2c & 2d).

File Name: Supplementary table 11.

Description: Cellular cholesterol following ZNF529 silencing (Figure 2f).

File Name: Supplementary table 12.

Description: Comparison of protein altering variants with related traits.

File Name: Supplementary table 13.

Description: Gene based test results.

File Name: Supplementary table 14.

Description: Burden test for APOB LoF variants and liver-related traits in the HUNT study.

File Name: Supplementary table 15.

Description: PheWAS results in UK biobank for all protein-altering variants that were able to be imputed from the TOPMed imputation panel. 1,342 ICD based trait codes were examined for association with any of the variants, only traits with at least 1 significant association (P < 3.5x10-5) are shown.

File Name: Supplementary table 16.

Description: Genes selected for designing putative nonsense codons based on prior knowledge of their involvement in Alzheimer's, cardiovascular or metabolic disease or for their inclusion in the ACMG 56 genes.

File Name: Supplementary table 17.

Description: Selection of variants for inclusion in custom content for the Hunt-Michigan Illumina Infinium Human CoreExome array.

File Name: Supplementary table 18.

Description: Genomic control in liver traits assessed in the SardiNIA Project.

File Name: Supplementary table 19.

Description: Primer pairs used for qPCR analyses.
