# Supplementary material for: Loss-of-function genomic variants highlight potential therapeutic targets for cardiovascular disease
Source: Nat Commun. 2020 Dec 18;11:6417. doi: 10.1038/s41467-020-20086-3 (PMC7749177; doi:10.1038/s41467-020-20086-3)
Supplement: Supplementary file 5 — Reporting Summary [file 41467_2020_20086_MOESM5_ESM.pdf]

nature research

Corresponding author(s): Jonas B Nielsen
Cristen J Willer
Kristian Hveem
Y. Eugene Chen

Last updated by author(s): Jul 24, 2020

Reporting Summary

Nature Research wishes to improve the reproducibility of the work that we publish. This form provides structure for consistency and transparency in reporting. For further information on Nature Research policies, see Authors & Referees and the Editorial Policy Checklist.

Statistics

For all statistical analyses, confirm that the following items are present in the figure legend, table legend, main text, or Methods section.

n/a	Confirmed	
☐	☒	The exact sample size (*n*) for each experimental group/condition, given as a discrete number and unit of measurement
☐	☒	A statement on whether measurements were taken from distinct samples or whether the same sample was measured repeatedly
☐	☒	The statistical test(s) used AND whether they are one- or two-sided<br>*Only common tests should be described solely by name; describe more complex techniques in the Methods section.*
☐	☒	A description of all covariates tested
☐	☒	A description of any assumptions or corrections, such as tests of normality and adjustment for multiple comparisons
☐	☒	A full description of the statistical parameters including central tendency (e.g. means) or other basic estimates (e.g. regression coefficient) AND variation (e.g. standard deviation) or associated estimates of uncertainty (e.g. confidence intervals)
☐	☒	For null hypothesis testing, the test statistic (e.g. *F*, *t*, *r*) with confidence intervals, effect sizes, degrees of freedom and *P* value noted<br>*Give P values as exact values whenever suitable.*
☒	☐	For Bayesian analysis, information on the choice of priors and Markov chain Monte Carlo settings
☒	☐	For hierarchical and complex designs, identification of the appropriate level for tests and full reporting of outcomes
☐	☒	Estimates of effect sizes (e.g. Cohen's *d*, Pearson's *r*), indicating how they were calculated

Our web collection on statistics for biologists contains articles on many of the points above.

Software and code

Policy information about availability of computer code

Data collection	In vitro studies: Western blots were visualized and quantified using LI-COR Biosciences Software, version 2.1. Fluorescence microscopy data were collected using Olympus DP Controller Software version 3.3.1.292, images were merged using ImageJ 1.52a software (NIH).
Data analysis	If not otherwise specified, statistical tests were performed using R (v.3.6.0). Imputation was performed using Minimac3. Summary statistics from SardiNIA and Biobank Japan were in Human Genome Build hg19, the positions were mapped to Human Genome Build hg38 using liftOver (v.2018-03-13). Genomic association tests were performed using SAIGE or EMMAX as implemented in EPACTS. Meta-analyses were conducted using METAL (v.2011-03-25). Exome-wide gene-based SKAT-O tests were performed using SAIGE-GENE (v36). All variants were annotated using ANNOVAR (v.2018-04-16). RNA sequence alignment and processing was done using Tophat2 (v2.0.13 11/5/19 7:29:00 PM), Samtools (v1.9), and bedtools (v.2.22.0). Data analyses of in vitro studies were performed using SPSS (v.24.0).

For manuscripts utilizing custom algorithms or software that are central to the research but not yet described in published literature, software must be made available to editors/reviewers. We strongly encourage code deposition in a community repository (e.g. GitHub). See the Nature Research guidelines for submitting code & software for further information.

Data

Policy information about availability of data

All manuscripts must include a data availability statement. This statement should provide the following information, where applicable:

- Accession codes, unique identifiers, or web links for publicly available datasets
- A list of figures that have associated raw data
- A description of any restrictions on data availability

Genome-wide summary statistics will be deposited upon manuscript acceptance. The raw RNA sequence reads are available for download at: https://www.ncbi.nlm.nih.gov/bioproject/PRJNA549711/.

Field-specific reporting

Please select the one below that is the best fit for your research. If you are not sure, read the appropriate sections before making your selection.

☒ Life sciences ☐ Behavioural & social sciences ☐ Ecological, evolutionary & environmental sciences

For a reference copy of the document with all sections, see nature.com/documents/nr-reporting-summary-flat.pdf

Life sciences study design

All studies must disclose on these points even when the disclosure is negative.

Sample size	Genomic studies: The sample size of this study was not predetermined. We included as many study participants as possible to improve power for discovering novel genetic associations.
Data exclusions	In vitro studies: No data were excluded from the analyses.
Replication	In vitro studies: findings from cell culture studies were replicated in at least 3 biologically distinct samples. All attempts at replication were successful.
Randomization	No group allocation was performed in this study.
Blinding	Genomic studies: Blinding was not relevant to this observational study. Participants were analyzed according to levels of biomarkers or observed disease status.<br>In vitro studies: RNA sequencing was performed by technicians blinded to cell treatments. Some qPCRs were performed by a technician blinded to cell treatments and others were performed by investigators not blinded to cell treatments. All experiments consistently indicated suppression of ZNF529 and up-regulation of LDLR, regardless of blinding. Random fields were chosen and photographed in a blinded fashion for DiI-LDL uptake studies.

Reporting for specific materials, systems and methods

We require information from authors about some types of materials, experimental systems and methods used in many studies. Here, indicate whether each material, system or method listed is relevant to your study. If you are not sure if a list item applies to your research, read the appropriate section before selecting a response.

Materials & experimental systems

n/a	Involved in the study	
☐	☒	Antibodies
☐	☒	Eukaryotic cell lines
☒	☐	Palaeontology
☒	☐	Animals and other organisms
☐	☒	Human research participants
☒	☐	Clinical data

Methods

n/a	Involved in the study	
☒	☐	ChIP-seq
☒	☐	Flow cytometry
☒	☐	MRI-based neuroimaging

Antibodies

Antibodies used	Anti-LDL Receptor [EP1553Y] (ab52818), Abcam. Anti-β-Actin [8H10D10] (#3700), Cell Signaling Technology. IRDye 800CW secondary antibodies: donkey anti-Rabbit IgG (926-32213) and donkey anti-mouse (926-68072), LI-COR Biotechnology.
Validation	Validation statements by the manufacturers: ab52818 has been validated in Western blot, tested in human and mouse samples including HepG2 cells, and referenced in 71 publications (https://www.abcam.com/ldl-receptor-antibody-ep1553y-ab52818.html, 7-24-20). 8H10D10 has been validated in Western blot, tested in various cell types, including human and mouse cells and referenced in 1453 publications (https://www.cellsignal.com/products/primary-antibodies/b-actin-8h10d10-mouse-mab/3700, 7-24-20).

Eukaryotic cell lines

Policy information about cell lines

Cell line source(s)	The HepG2 human hepatoma cell line was obtained from the American Type Culture Collection (ATCC, HB-8065).
Authentication	HepG2 cells express known hepatocyte markers, including albumin, and also express the LDL receptor. The cell line used was not authenticated.

Mycoplasma contamination	Cells were not tested for Mycoplasma contamination.
Commonly misidentified lines (See ICLAC register)	No commonly misidentified cell lines were used in the study.

Human research participants

Policy information about studies involving human research participants

Population characteristics	More that 600,000 participants were recruited at four population studies (HUNT, SardiNIA, Biobank Japan and UK Bioban). All participants were older than 18 years of age, except for the SardiNIA study where participants were >14 years of age. Both sexes were included. All patients gave written informed consent. All studies was conducted in accordance with the Declaration of Helsinki.
Recruitment	People from the general population were invited to participate. This might have resulted in 'healthy responder bias'.
Ethics oversight	The following originations approved study protocols: The Data Inspectorate and the Regional Ethics Committee for Medical Research in Norway. University of Michigan, USA. Istituto di Neurogenetica e Neurofarmacologia, Italy. MedStar Research Institute, United States.

Note that full information on the approval of the study protocol must also be provided in the manuscript.
