# Supplementary material for: Loss-of-function genomic variants highlight potential therapeutic targets for cardiovascular disease
Source: Nat Commun. 2020 Dec 18;11:6417. doi: 10.1038/s41467-020-20086-3 (PMC7749177; doi:10.1038/s41467-020-20086-3)
Supplement: Supplementary file 2 — Peer Review File [file 41467_2020_20086_MOESM2_ESM.docx]

**SUPPLEMENTARY MATERIALS**

*‘**Loss-of-function genomic variants highlight potential therapeutic targets for cardiovascular disease’* by Nielsen J.B., Rom O. et al., Nature Communications 2020.

**SUPPLEMENTARY TABLES (IN SEPARATE EXCEL FILE)**

**Supplementary table 1.** Sample sizes for 9 liver traits examined in the HUNT study.

**Supplementary table 2.** 271 unique index variants (P < 5x10^-8^) for one or more traits within 201 loci (representing 329 significant variant-trait combinations).

**Supplementary table 3**. 161 unique variant-trait associations with p-value < 5x10-8 identified by conditional analysis of the 201 primary loci.

**Supplementary table 4**. 351 unique variants identified from transethnic meta-analysis (426 variant-trait associations).

**Supplementary table 5**. Protein-altering variants identified from analysis of custom content.

**Supplementary table 6.** ZNF529 mRNA levels following its knockdown in HepG2 cells (Figure 3a)

**Supplementary table 7**. Genes with altered expression pattern following ZNF529 silencing in HepG2 cells.

**Supplementary table 8.** Pathway enrichment analysis using 476 differentially expressed genes.

**Supplementary table 9.** LDLR mRNA levels following ZNF529 silencing (Figure 3b)

**Supplementary table 10:** LDLR protein abundance following ZNF529 silencing (Figure 3c & 3d)

**Supplementary table 11.** Cellular cholesterol following ZNF529 silencing (Figure 3f)

**Supplementary table 12**. Comparison of protein altering variants with related traits.

**Supplementary table 13**. Gene-based test results.

**Supplementary table 14**. Burden test for APOB LoF variants and liver-related traits in the HUNT study.

**Supplementary table 15.** PheWAS results in UK biobank for all protein-altering variants that were able to be imputed from the TOPMed imputation panel. 1,342 ICD based trait codes were examined for association with any of the variants, only traits with at least 1 significant association (P < 3.5x10-5) are shown.

**Supplementary table 16**: Genes selected for designing putative nonsense codons based on prior knowledge of their involvement in Alzheimer's, cardiovascular or metabolic disease or for their inclusion in the ACMG 56 genes.

**Supplementary table 17**. Selection of variants for inclusion in custom content for the Hunt-Michigan Illumina Infinium Human CoreExome array.

**Supplementary table 18**. Genomic control in liver traits assessed in the SardiNIA Project.

**Supplementary table 19**. Primer pairs used for qPCR analyses.

**SUPPLEMENTARY FIGURES**

***Supplementary figure 1 – Study design.***

Approximately 70,000 individuals from the HUNT study have been genotyped and have available biomarker data. Included in the genotyping array for these individuals were custom content variants selected based on variants identified from whole-genome sequencing of Norwegians, known variants associated with lipid phenotypes, and predicted stop codons in ACMG genes. We analyzed the genotyped variants directly for association with 9 liver-related phenotypes, analyzed the TOPMed imputed results, and performed trans-ethnic meta-analysis with two additional cohorts. MI; myocardial infarction, WGS; whole genome sequencing, ICD; international classification of diseases, SAIGE; Scalable and Accurate Implementation of GEneralized mixed model.

***Supplementary figure 2 – QQ plots from HUNT analyses.***

Markers are stratified by minor allele frequency below vs. above 0.01. Dots indicate observed P-values (­–log_10_[P-value]) compared with those expected by chance under the null hypothesis (no association). The black line indicates the identity (no association) with corresponding 95% confidence intervals.

***Supplementary figure 3 – QQ plots from meta-analyses.***

Markers are stratified by minor allele frequency below vs. above 0.01. Dots indicate observed P-values (­–log_10_[P-value]) compared with those expected by chance under the null hypothesis (no association). The black line indicates the identity (no association) with corresponding 95% confidence intervals.

***Supplementary figure 4 – Genome-wide significant results generated by each methodology.***

*The number of index variants that reached genome-wide significance are indicated on the Venn diagram by the four different methodological approaches and their overlap. The black numbers indicate the index variants, white indicates index variants that are coding variants, and the number in brackets indicates index variants that are LoF.*

***Supplementary figure 5 – Association pattern for variants in proximity to ZNF529 that were associated with one or more traits at a genome-wide significance level (P<5x10^-8^).***

*Beta is reported with respect to 1 SD change (LDL-C, TC, ALT, ALP, AST, CRP, GGT) or disease status (MI, CAD, hypertension). *Variant completely independent with p.K(405/423/318)X (r^2^ <0.001). **Variant identified through conditional analyses and current association analysis is conditioned on chr19:35981141:C:G. The variant itself is completely independent with p.K(405/423/318)X (r^2^ <0.001).*

***Supplementary figure 6 – A distinct gene expression pattern in HepG2 cells following ZNF529 knockdown.***

*HepG2 cells were transfected with siRNA targeting ZNF529 (siZNF529, orange dots, N=4) or non-targeting siRNA control (siCTL, green dots, N=4 biologically independent samples). Following 48h, RNA was purified from the cells and used for library preparation and sequencing on a NovaSeq 6000 System. Principal component analysis is shown underlining a distinct gene expression pattern in HepG2 cells transfected with siZNF529 vs. siCTL.*

***Supplementary figure 7 – Dose-dependent saturation of DiI-LDL uptake.***

*HepG2 cells were incubated for 5 h with increasing levels of DiI-LDL (0-25 μg/ml) in serum-free DMEM. Nuclei were stained with DAPI 30 min prior to visualization by fluorescent microscopy (N=3* *biologically independent samples). DiI-LDL was taken-up by the cells in a dose-dependent manner, resulting in saturated uptake at 25 μg/ml (scale bars=200 µm). Source data are provided as a Source Data file.*

***Supplementary figure 8 – Competitive binding between DiI-labeled and unlabeled LDL.***

*To assess the specificity of the binding, HepG2 cells were incubated for 5 h with DiI-LDL (10 or 25 μg/ml) in serum-free DMEM with or without pretreatment with 25-fold excess amounts of unlabeled LDL (250 or 625 μg/ml). Nuclei were stained with DAPI 30 min prior to visualization by fluorescent microscopy (N=3 biologically independent samples). The uptake of DiI-LDL was inhibited in the presence of 25-fold excess amounts of unlabeled LDL (scale bars=200 µm). Source data are provided as a Source Data file.*

***Supplementary figure 9 – Confirmation of ZNF529:p.K(405/423/318)X using Sanger sequencing.***

*The figure shows the raw chromatograms from one control, one heterozygous carrier, and the only homozygous carrier.*

***Supplementary figure 10 – UK Biobank PheWAS results for all protein-altering variants.***

A total of 24 protein-altering variants were statistically significantly (P < 3.5x10^-5^) associated with one or more ICD code-defined diseases in UK Biobank. Arrows denote the direction of effect for the minor allele. Larger arrows signify more statistically significant associations. Statistically non-significant associations are not shown.
